# The Enzyme Glucose‐1‐Phosphate Thymidylyltransferase RmlA Plays a Crucial Role in the Pathogenesis of *Pectobacterium actinidiae* GX1

**DOI:** 10.1111/mpp.70118

**Published:** 2025-07-04

**Authors:** Zhixiang Yuan, Yijie Yu, Tingmi Yang, Yuwei Xue, Jiangfei Hu, Njoroge Hellen Wambui, Zhuang Liu, Mingzhao Wang, Hongxia Liu

**Affiliations:** ^1^ State Key Laboratory of Agricultural and Forestry Biosecurity Nanjing Agricultural University Nanjing China; ^2^ Changzhou Institute of Materia Medica Co., Ltd Changzhou China; ^3^ Guangxi Academy of Specialty Crops Guangxi Key Laboratory of Germplasm Innovation and Utilization of Specialty Commercial Crops in North Guangxi Guilin Guangxi China

**Keywords:** biosynthesis‐related gene, genome, pathogenicity, *Pectobacterium actinidiae*, polysaccharide

## Abstract

*Pectobacterium actinidiae* is one of the primary pathogens that causes summer canker disease in kiwifruit, yet its pathogenic mechanisms remain unknown. The exopolysaccharide PCAP‐1a, isolated from the fermentation broth of *P. actinidiae* strain GX1, exhibits notable cytotoxicity and acts as a virulence factor facilitating host infection. Genome‐wide analysis revealed a 21‐gene cluster responsible for the biosynthesis of exopolysaccharides in GX1. Homologous recombination was used to systematically knock out these genes, which led to the identification of RmlA as a key protein in the synthesis of the PCAP‐1a precursor. The deletion of the *rmlA* gene significantly affected the yield of PCAP‐1a and resulted in a direct reduction in GX1 pathogenicity. Further studies revealed that mutations in the substrate binding site of RmlA weakened its capacity to bind G‐1‐P and dTTP, which led to markedly reduced pathogenicity in the corresponding complemented strains. This study indicates that the exopolysaccharide PCAP‐1a serves as a virulence factor in the pathogenesis of GX1, and its biosynthesis depends on the polysaccharide synthesis gene *rmlA* and the substrate binding activity of its encoded protein.

## Graphical abstract

Deletion of the *rmlA* gene negatively affected PCAP‐1a yield, structure and directly diminished GX1 pathogenicity. Furthermore, mutations within RmlA's substrate binding site impaired its ability to bind G‐1‐P and dTTP.

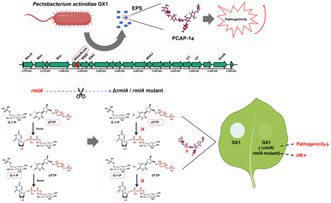

## 1 Introduction

Kiwifruit vines can be infected by a variety of pathogens during their growth cycle (Tao et al. 2022). In recent years, a form of kiwifruit canker, known as ‘kiwifruit summer canker’ and caused by *Pectobacterium actinidiae*, has emerged in South Korea and Guangxi, China. This disease is highly infectious and occurs mainly in the summer and autumn, posing a serious threat to the growth of kiwifruit vines. Kiwifruit summer canker affects mainly the branches, stems, buds, leaves and tender shoots of the plant and has little effect on the roots or fruits (Yan, Liu, et al. 2019; Wu et al. 2017). Upon infection, kiwifruit summer canker rapidly spreads from dim spots to irregular brown spots several millimetres in size on the leaves. The lesions on branches or stems are initially brown and watery, followed by red mucus exudation as the disease progresses, rapidly causing the withering of branches or whole plants, which not only severely affects the quality and yield of kiwifruit but also causes economic damage (Zhang et al. 2023). Owing to the relatively recent discovery of this disease, research on the pathogen remains limited, leaving its precise infection mechanism unclear.

Bacteria, through their potent biosynthetic capabilities, can synthesise a diverse array of polymers with unique chemical structures and various biological functions (Salimi and Farrokh 2023). These biopolymers can be categorised into two major classes on the basis of their localisation: intracellular biopolymers and extracellular biopolymers (Nwodo et al. 2012). The latter can be further subdivided into polysaccharides, inorganic polyanhydrides, polyesters and polyamides (Cerning 1995; Rehm 2010), among which polysaccharides are particularly known for their variety and diverse activities. The outer surface of the cell membrane of the majority of bacteria is enveloped by a layer of polysaccharides in a complex network of carbohydrate molecular chains (Kaur and Dey 2023). These polysaccharides not only form a viscous capsular substance covalently bound to the cell surface outside the cell wall/membrane (Ruas‐Madiedo et al. 2002) but also constitute a loose and sticky mucous layer that can adhere to the cell surface or be secreted into the external environment as exopolysaccharides (EPSs) (Schmid et al. 2015). Accumulating evidence demonstrates that EPSs consistently play a critical role in the pathogenic process of pathogens, with numerous studies confirming their function as virulence factors during host infection (Corsaro et al. 2001; Denny 1995; Espinosa et al. 2003; Goodman et al. 1974). For example, *Ralstonia solanacearum* produces large amounts of EPSs, which are among its most crucial virulence determinants. The quantity of EPSs produced by the pathogen can be indicative of the virulence of *R. solanacearum* (Saile et al. 1997). *Pectobacterium atrosepticum* EPS can suppress chitooligosaccharide‐induced immune responses and act as a virulence factor during infection (Islamov et al. 2021). EPSs from pathogens such as *Xanthomonas campestris* and *Pseudomonas aeruginosa* can act as virulence factors by chelating Ca^2+^ to prevent Ca^2+^ influx, thereby inhibiting the activation of the pattern‐triggered immunity (PTI) response in plants (Aslam et al. 2008). Recent studies have indicated that the EPS PCAP‐1a from *Pectobacterium actinidiae* promotes the development of host soft rot disease; interestingly, PCAP‐1a does not inhibit flg22‐induced immune responses (Yuan et al. 2024).

The aim of this study was to explore the relationship between the pathogenicity of *P. actinidiae* GX1 and its EPS PCAP‐1a and to functionally assess its key biosynthetic genes. By integrating bioinformatics with gene knockout and molecular interaction techniques, the critical role of the polysaccharide synthesis gene *rmlA* in the pathogenicity of *P. actinidiae* GX1 was determined.

## 2 Results

### 2.1 Analysis of the Polysaccharide Synthase Gene Cluster in *P. actinidiae* GX1

**FIGURE 1:**
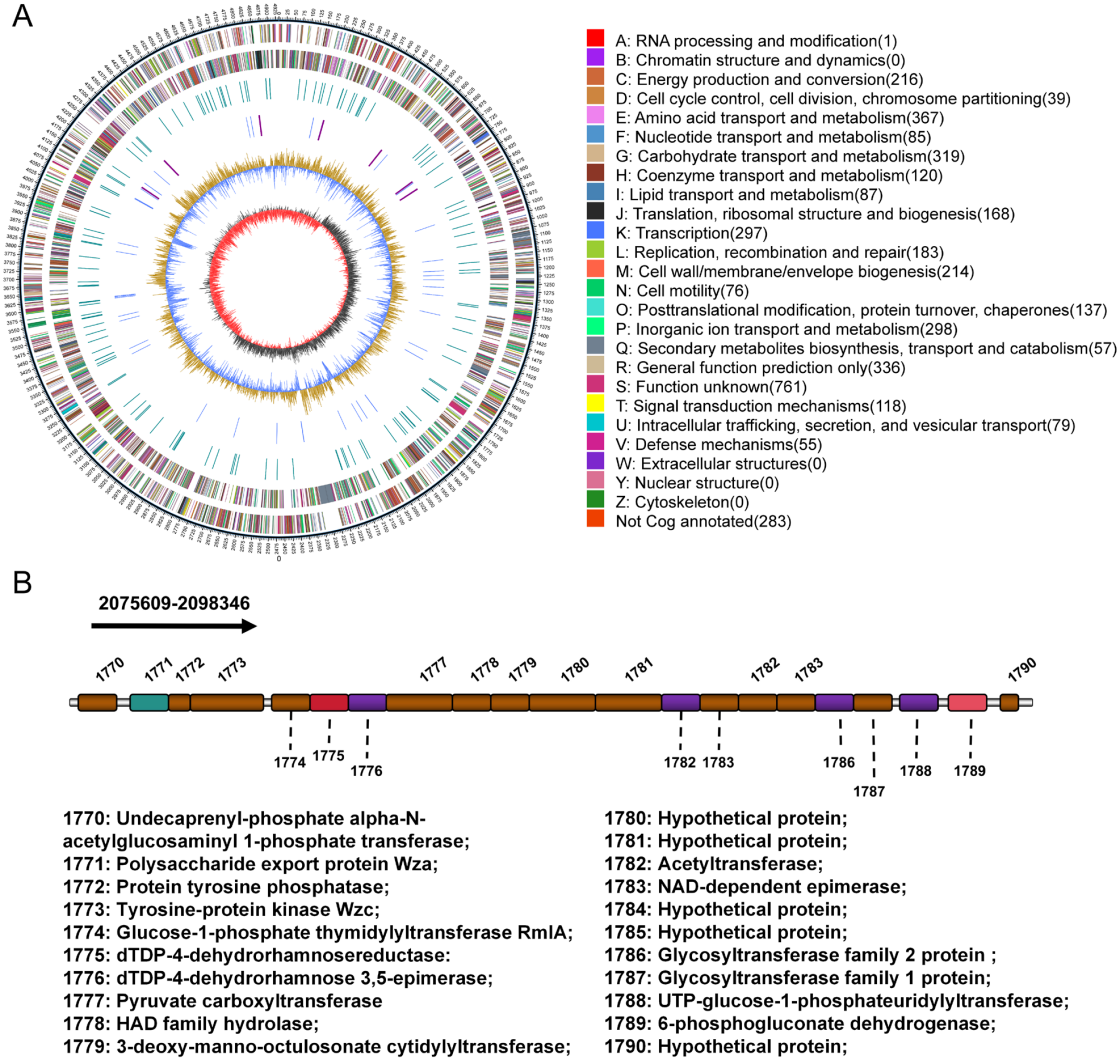
Characterisation of the complete genome map and exopolysaccharide (EPS) biosynthesis gene clusters of *Pectobacterium actinidiae* GX1. (A) Whole‐genome map of *P. actinidiae* GX1. The outer ring represents various functional annotations based on COG categories, with colour coding for different categories of biological processes. The centre of the map shows a graphical representation of the genomic features and annotations. (B) Analysis of EPS biosynthesis gene clusters in *P. actinidiae* GX1. The gene cluster diagram shows the specific genes involved in EPS biosynthesis. Each gene in the cluster is annotated with its corresponding function.

Unlike proteins directly encoded by genes, the production of exopolysaccharides starts with the reassembly and folding of sugar units catalysed by various enzymes that are encoded by gene clusters, and the successful synthesis of a polysaccharide is potentially determined and completed by dozens of genes (Sun and Zhang 2021). To elucidate the synthesis pathway of PCAP‐1a, we performed sequencing analysis of the whole genome of *P. actinidiae* GX1. As depicted in Figure 1A, the genome of *P. actinidiae* GX1 consists of a double‐stranded circular chromosome (4,935,467 bp in length) containing nine gene islands and seven prophages, with an average GC content of 51.58% (Figure 1A).

**FIGURE 2:**
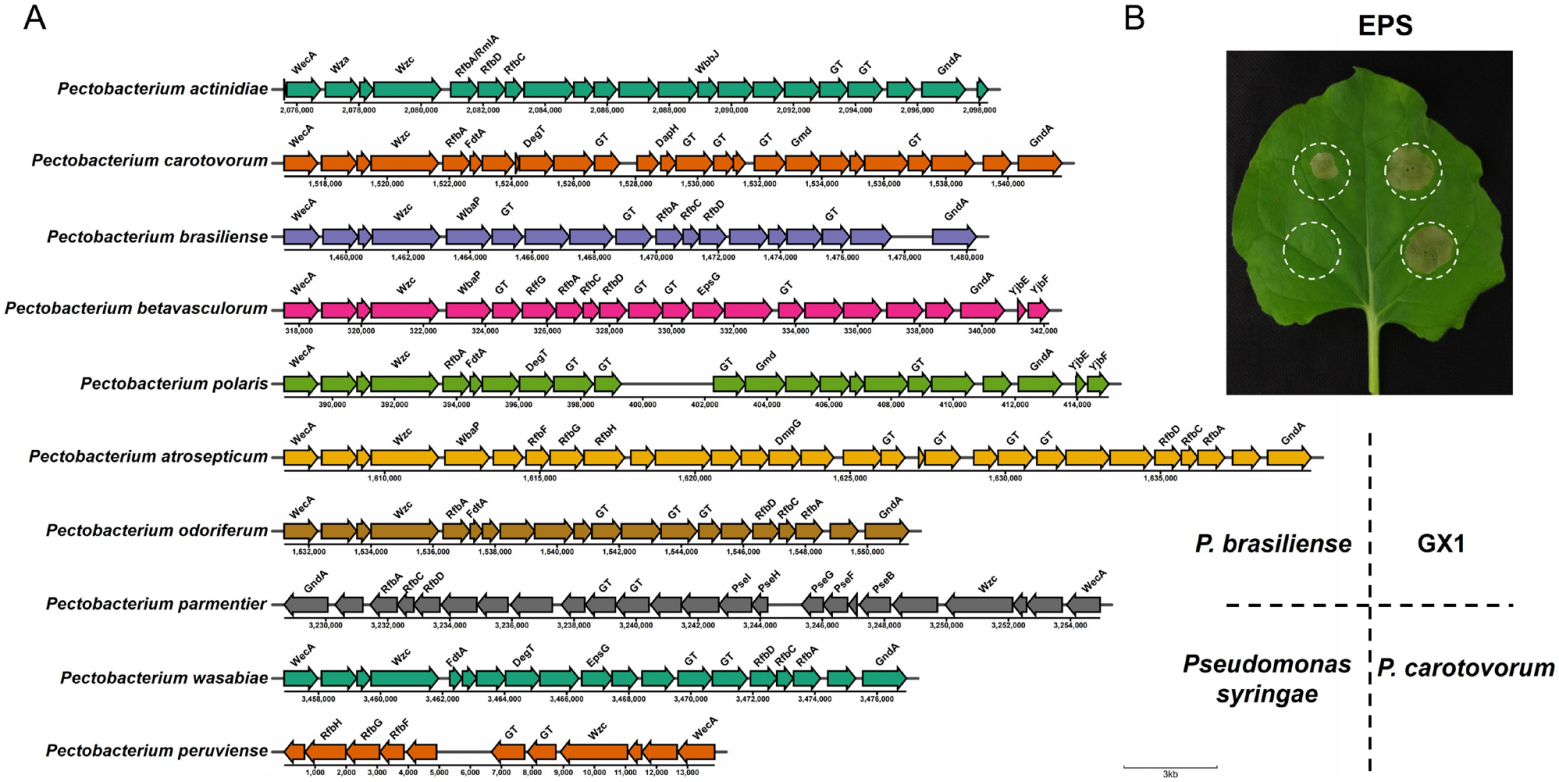
Analysis of exopolysaccharide (EPS) biosynthesis‐related gene clusters in the *Pectobacterium* genus. (A) Comparative analysis of EPS biosynthesis‐related gene clusters in the *Pectobacterium* genus. The clusters represent genes involved in the biosynthesis of EPS in various *Pectobacterium* species, key genes such as glycosyltransferases (GTs). These clusters have been aligned to show their relative relatedness, highlighting conserved and divergent regions across species. (B) Biological activity tests of the exopolysaccharides of GX1, *P. brasiliense* 1692, *P. carotovorum* WPP14 and *Pseudomonas syringae* Susan2139. The resulting EPSs were extracted and prepared as 0.5 mg/mL solutions, which were then infiltrated into the same *Nicotiana benthamiana* leaf. Cell death in each leaf was observed after 72 h, and the observed phenotypes correlate with the presence or absence of specific biosynthesis‐related genes in (A).

The synthesis of exopolysaccharides in prokaryotic microorganisms involves a constellation of genes encoding enzymes and regulatory factors that collectively contribute to the biosynthesis and modification of EPSs (Sun and Zhang 2021). This genomic repertoire commonly encompasses genes encoding glycosyltransferases, epimerases, dehydrogenases, reductases, glycosyl transport proteins and various regulatory elements (Saulnier et al. 2011). The genetic composition and combination order of EPS synthesis genes can vary among different bacterial strains, resulting in diversity in the structure and properties of bacterial EPSs (Sun and Zhang 2021). The EPS synthesis gene clusters of several members of the genus *Pectobacterium* are illustrated in Figure 1B. Whole‐genome analysis revealed that the exopolysaccharide synthesis gene cluster of *P. actinidiae* GX1 comprises 21 genes, with a combined length of 22,727 bp. The primary genes involved in EPS synthesis include those encoding polysaccharide transport proteins (WecA, Wza, Wzc), glucose phosphate thymidylyltransferase, epimerases and glycosyltransferases. Using genomic data, we observed that the EPS synthesis gene clusters of *P. carotovorum* and *P. brasiliense* share a high degree of similarity with that of GX1 (Figure 2A). The crude EPSs were extracted from *P. carotovorum* and *P. brasiliense* and then infiltrated into *Nicotiana benthamiana* leaves, and both induced cell death in *N. benthamiana* leaf cells, whereas EPSs from *Pseudomonas syringae* did not prompt this response (Figure 2B). These findings suggest that the EPSs from *P. carotovorum* or *P. brasiliense* may share structural similarities with PCAP‐1a from GX1.

### 2.2 Knockout of the EPS Synthesis Genes in *P. actinidiae* GX1

Some of the key genes within this cluster shown in Figure 2B were individually knocked out via homologous recombination and biparental mating, and the pEX18 plasmid with Km‐lox segment and the S17–λpir strain were used to promote large‐scale gene exchange to knock out several key genes within this cluster. The resulting mutant strains were then used to infect *N. benthamiana* leaves. By analysing cell death under different treatments, we preliminarily identified key genes associated with the pathogenicity of GX1 and the synthesis of EPSs. Interestingly, among all the knockout strains, only the deletion of gene 1774 reduced the ability of *P. actinidiae* GX1 to induce cell death (Figure S1). Gene 1774 encodes the enzyme glucose‐1‐phosphate thymidylyltransferase RmlA, which is known to catalyse the conversion of glucose‐1‐phosphate (G‐1‐P) and dTTP into dTDP‐glucose, a precursor commonly involved in bacterial sugar metabolism pathways that serves as a precursor for deoxyglucose (Harathi et al. 2016; Zheng et al. 2022). In many bacteria, deoxyglucose is a crucial component of EPSs, lipopolysaccharides and other polysaccharides within the cell wall (Azimi et al. 2020; Hirmondó et al. 2022; Silva et al. 2005). Given the critical role of RmlA in the biosynthesis of dTDP‐glucose, understanding its functional and structural properties is essential for elucidating its involvement in bacterial cell wall synthesis and its potential as a target for novel antimicrobial strategies.

### 2.3 The Impact of RmlA on the Pathogenicity of *P. actinidiae* GX1

**FIGURE 3:**
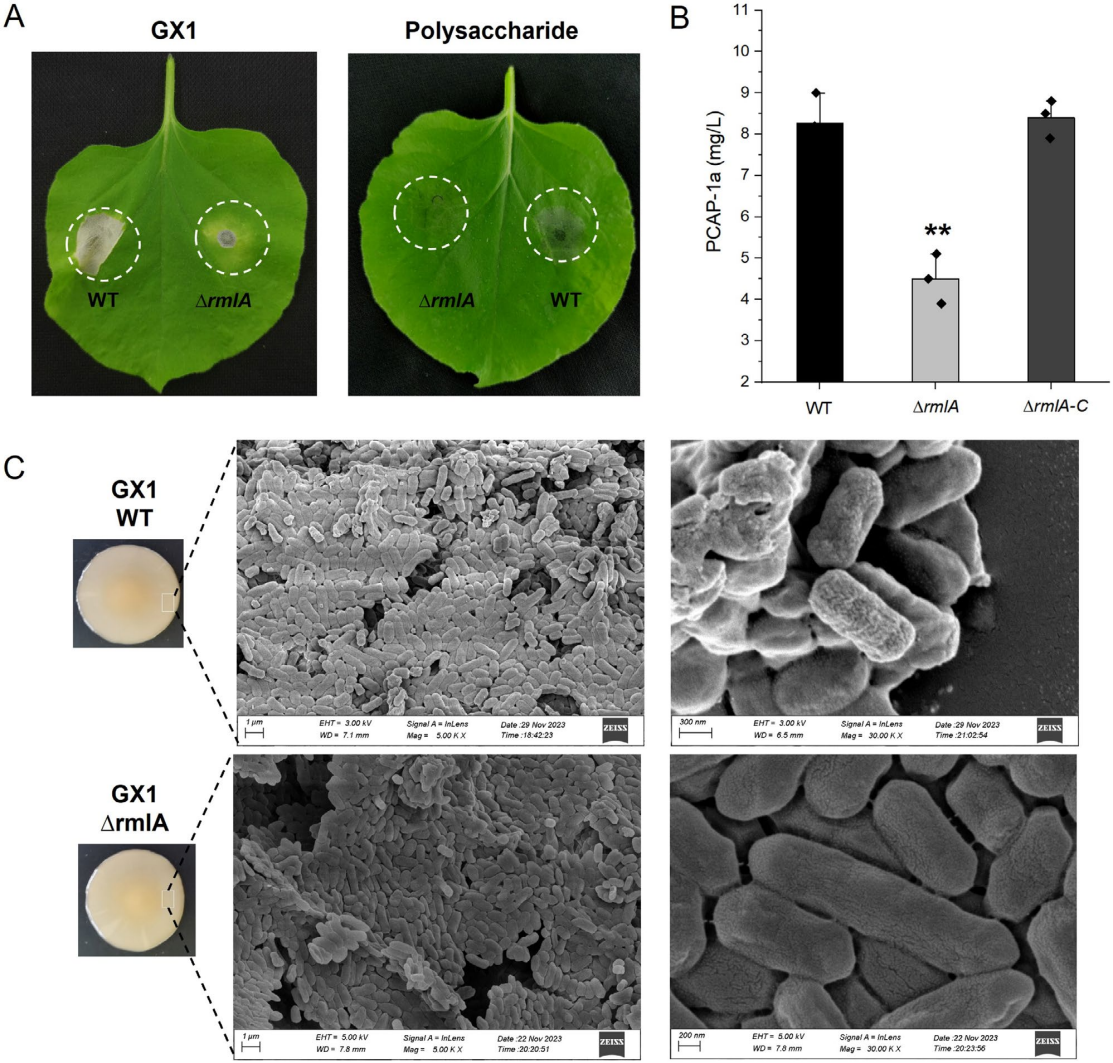
Impact of the *rmlA* gene on the yield of exopolysaccharides (EPSs) from and the morphology of *Pectobacterium actinidiae* GX1. (A) Both the *∆rmlA* strain and its EPSs had a reduced ability to induce *Nicotiana benthamiana* cell death. (B) The yield of EPSs produced by the *∆rmlA* strain decreased. The error bars represent the standard deviation; *n* = 3 bacterial samples. Significant differences from ANOVA are shown: ** = *p * < 0.01. The control is the amount of EPS produced by the wild‐type strain of GX1 (WT). The experiment was repeated three times, and the results all showed the same trend. (C) The colony morphology phenotypes of the WT and *∆rmlA* strains, as well as the surface morphology of the WT and *∆rmlA* strains, were observed through scanning electron microscopy.

**FIGURE 4:**
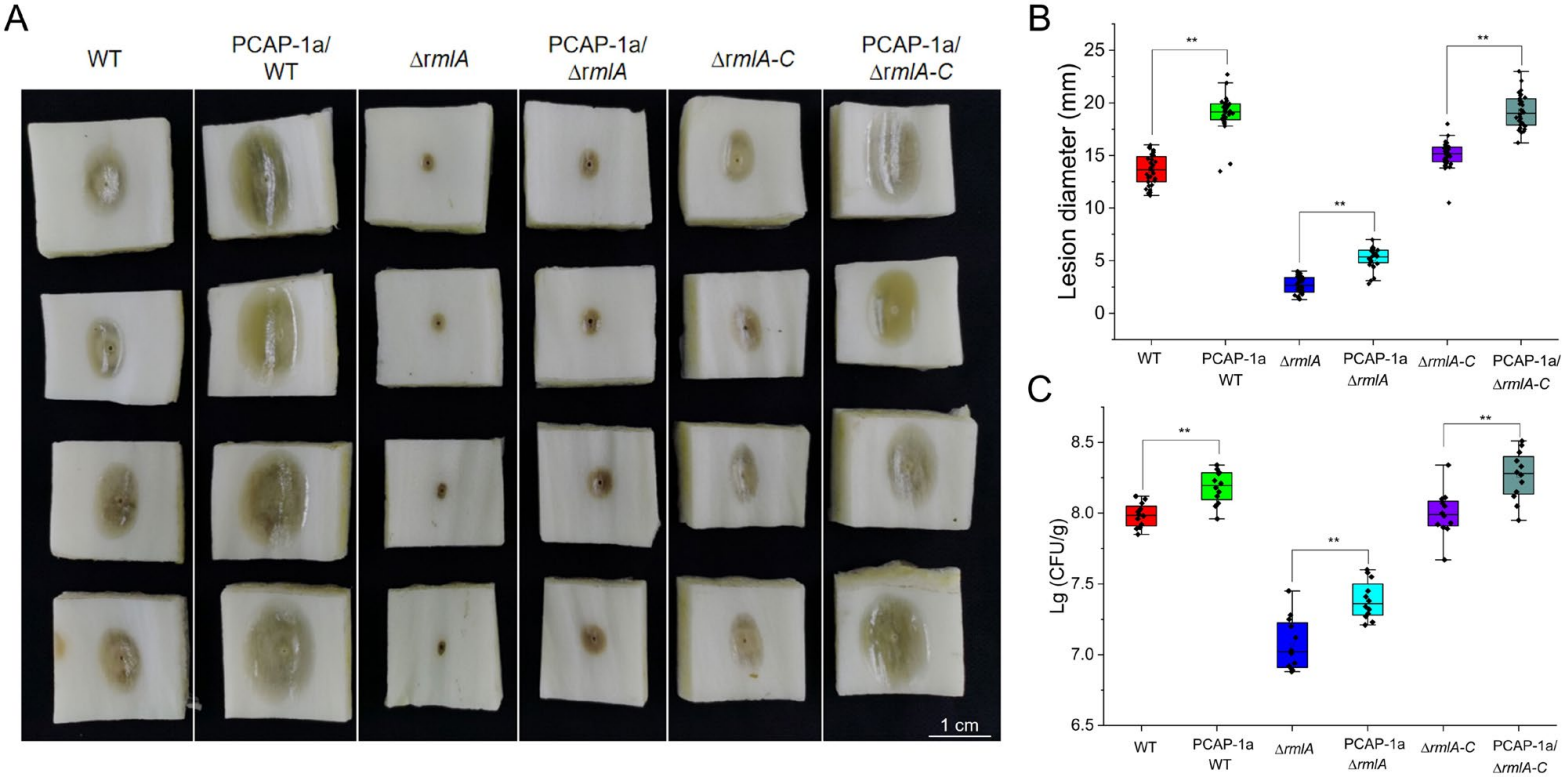
Impact of the *rmlA* gene on the pathogenicity of *Pectobacterium actinidiae* GX1. (A) To assess the pathogenicity of the ∆*rmlA* strain, *∆rmlA*, *∆rmlA‐C* and wild‐type (WT) strains were cultured to logarithmic growth stage, and the OD_600_ was adjusted to 0.4 with sterile water, and 6 μM PCAP‐1a was used for the treatment. Then, a 1.5 μL bacterial suspension or mixed solution of bacterial suspension and PCAP‐1a was inoculated on a Chinese cabbage core approximately 2 × 2 cm in size, and the lesion diameter and degree of bacterial colonisation were measured after 18 h at 28°C. (B) Lesion diameters corresponding to the assay data in (A). (C) Bacterial colonisation corresponding to the assay data in (A). The error bars represent the standard deviation; *n* = 12 stems. Significant differences from ANOVA are shown: ** = *p *< 0.01. The control consisted of Chinese cabbage stems incubated with WT GX1. The experiment was repeated three times, and the results all showed the same trend.

Given the substantial phenotypic alterations observed in the ∆*rmlA* strain, we hypothesised that the deletion of *rmlA* may affect the synthesis of the strain's EPSs. We extracted the EPS PCAP‐1a_*∆rmlA*_ from the *∆rmlA* fermentation broth. Compared with that of the wild‐type (WT) strain, the yield of PCAP‐1a_∆*rmlA*_ was reduced by approximately 45.5%. Conversely, the complemented strain with the reintroduced *rmlA* (∆*rmlA‐C*) showed no significant difference in PCAP‐1a yield compared to the WT strain (Figure 3B), suggesting that *rmlA* deletion restricts PCAP‐1a synthesis. Furthermore, the ability of the *∆rmlA* strain to induce cell death in *N. benthamiana* was reduced, suggesting that the disruption of *rmlA* may compromise the structural integrity of PCAP‐1a (Figure 3A). Notably, despite impaired EPS synthesis in the *∆rmlA* strain, its surface morphology was not significantly different from that of the WT strain according to scanning electron microscopy (Figure 3C). Pathogenicity assays on Chinese cabbage revealed that, compared with the WT strain, the *∆rmlA* mutant strain produced lesions with significantly smaller diameters and exhibited reduced bacterial colonisation (Figure 4A–C). The virulence of the *∆rmlA* mutant strain was partially restored following cotreatment with 6 μM PCAP‐1a produced by the WT strain. In contrast, the virulence of the *rmlA*‐complemented strain *∆rmlA‐C* was fully restored to the level of the WT strain (Figure 4A–C). To further elucidate the effects of PCAP‐1a on the adhesiveness of GX1, a GFP‐tagged vector was introduced into the bacterial strains by biparental mating. Following spray inoculation on *N. benthamiana* leaves, colonisation of the leaf surface was observed under a confocal microscope. The colonisation ability was evaluated by observing the area of green fluorescence. Consistent with the pathogenicity results, a significant reduction in colonisation ability was noted for the mutant strain *∆rmlA*, while the adhesion of the complemented strain was restored to levels comparable to those of the WT strain (Figure S2). Additionally, the presence of PCAP‐1a was found to increase or restore the colonisation levels to some extent (Figure S3). These findings suggest that *rmlA* may affect the pathogenicity of GX1 by influencing the synthesis of EPSs.

### 2.4 Prediction and Mutation of the RmlA Active Site

**FIGURE 5:**
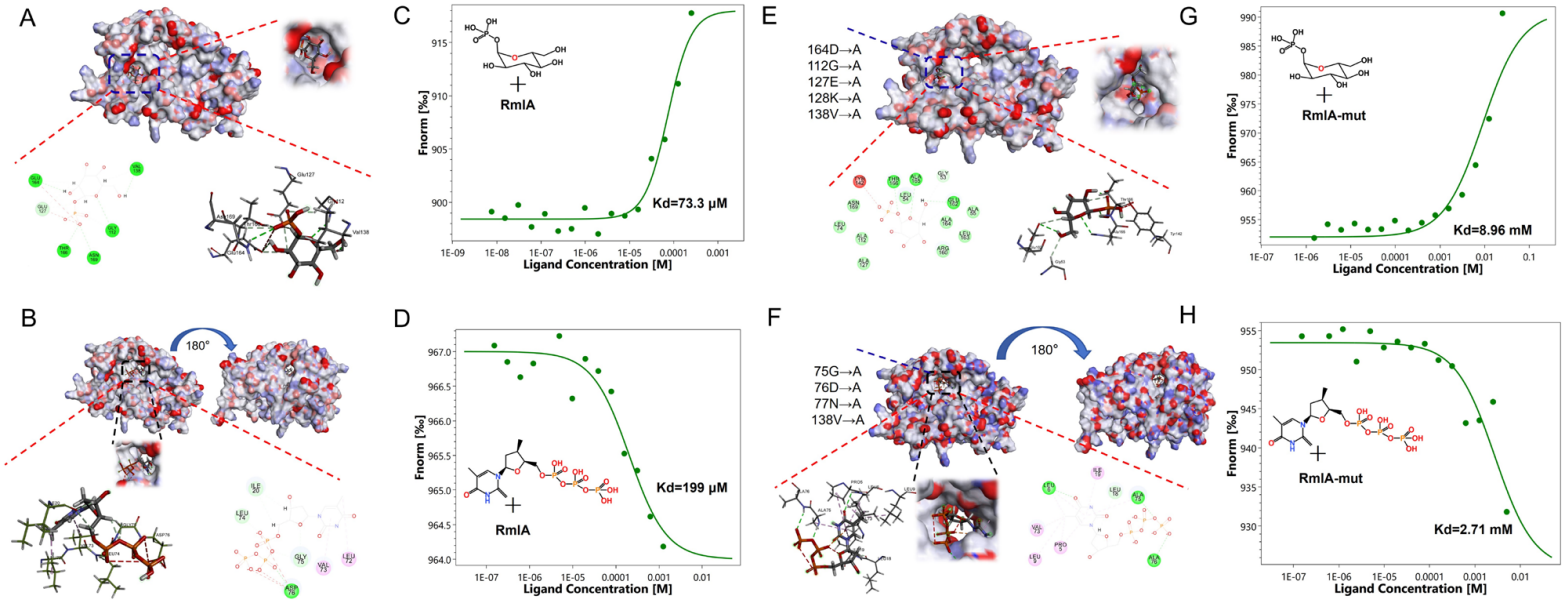
Heterologous expression of RmlA and its binding affinity for its substrates glucose‐1‐phosphate (G‐1‐P) and dTTP. (A) Molecular docking simulation of the RmlA binding site with G‐1‐P. (B) Molecular docking simulation of the RmlA binding site with dTTP. (C) Microscale thermophoresis (MST) was used to assess the binding affinity between RmlA and G‐1‐P. (D) MST was used to assess the binding affinity between RmlA and dTTP. (E–H) Binding affinity assays for RmlA mutants with substrates (G‐1‐P, dTTP). (E) Molecular docking simulations of RmlA mutant binding sites with G‐1‐P. (F) Molecular docking simulations of RmlA mutant binding sites with dTTP. (G) MST assays of the binding affinity between the RmlA mutant and G‐1‐P. (H) MST assays of the binding affinity between the RmlA mutant and dTTP.

The primary known biological function of RmlA is catalysis of the initial reaction for the synthesis of dTDP‐L‐Rha, which primarily involves the utilisation of G‐1‐P and dTTP to produce dTDP‐glucose, the latter of which frequently serves as a precursor for L‐rhamnose and deoxyglucose (Azimi et al. 2020; Hirmondó et al. 2022; Silva et al. 2005). In this study, we used the Phyre2 web portal to model the crystal structure of RmlA from GX1 and used Discovery Studio software to predict the corresponding binding sites, as shown in Figure 5A,B. According to the docking model of RmlA with G‐1‐P, G‐1‐P is located within a pocket of RmlA, where residues Glu164, Gly112, Glu127 and Val138 from RmlA primarily form hydrogen bonds or carbon–hydrogen bonds with the oxygen atoms or hydroxyl groups of G‐1‐P (Figure 5A). According to the docking model of RmlA with dTTP, dTTP is positioned inside the cavity of RmlA, with residues Gly75, Asp76 and Asn77 from RmlA mainly forming hydrogen bonds or carbon–hydrogen bonds with the oxygen atoms or phosphate groups of dTTP (Figure 5B). A mutated RmlA protein with mutations introduced at the aforementioned binding sites was obtained using a heterologous expression system (Figure S4). The crystal model of RmlA, after the key amino acid sites mentioned above were mutated, was obtained in the same manner. The mutated model was then used to interact with G‐1‐P and dTTP separately using Discovery Studio software. As shown in Figure 5E,F, the binding affinity of the mutated RmlA with G‐1‐P and dTTP was significantly reduced. The pocket‐like structure in RmlA that binds G‐1‐P could no longer be maintained, and Ala failed to form stable bonds with either G‐1‐P or dTTP. The binding affinity of RmlA for its substrates G‐1‐P and dTTP was verified using microscale thermophoresis (MST), as depicted in Figure 5C,D. Following ligand gradient dilution, both mutant and WT RmlA could bind to G‐1‐P and dTTP. However, there was a significant difference in binding affinity before and after mutation. Notably, the binding affinities of the mutated RmlA proteins were substantially altered: the affinity for G‐1‐P decreased by approximately 100‐fold, whereas the affinity of the mutant protein for dTTP was reduced to less than a tenth of that observed for the nonmutated proteins in Figure 5G,H.

### 2.5 The Substrate Binding Capacity of RmlA Affects the Pathogenicity of the Host Strain

**FIGURE 6:**
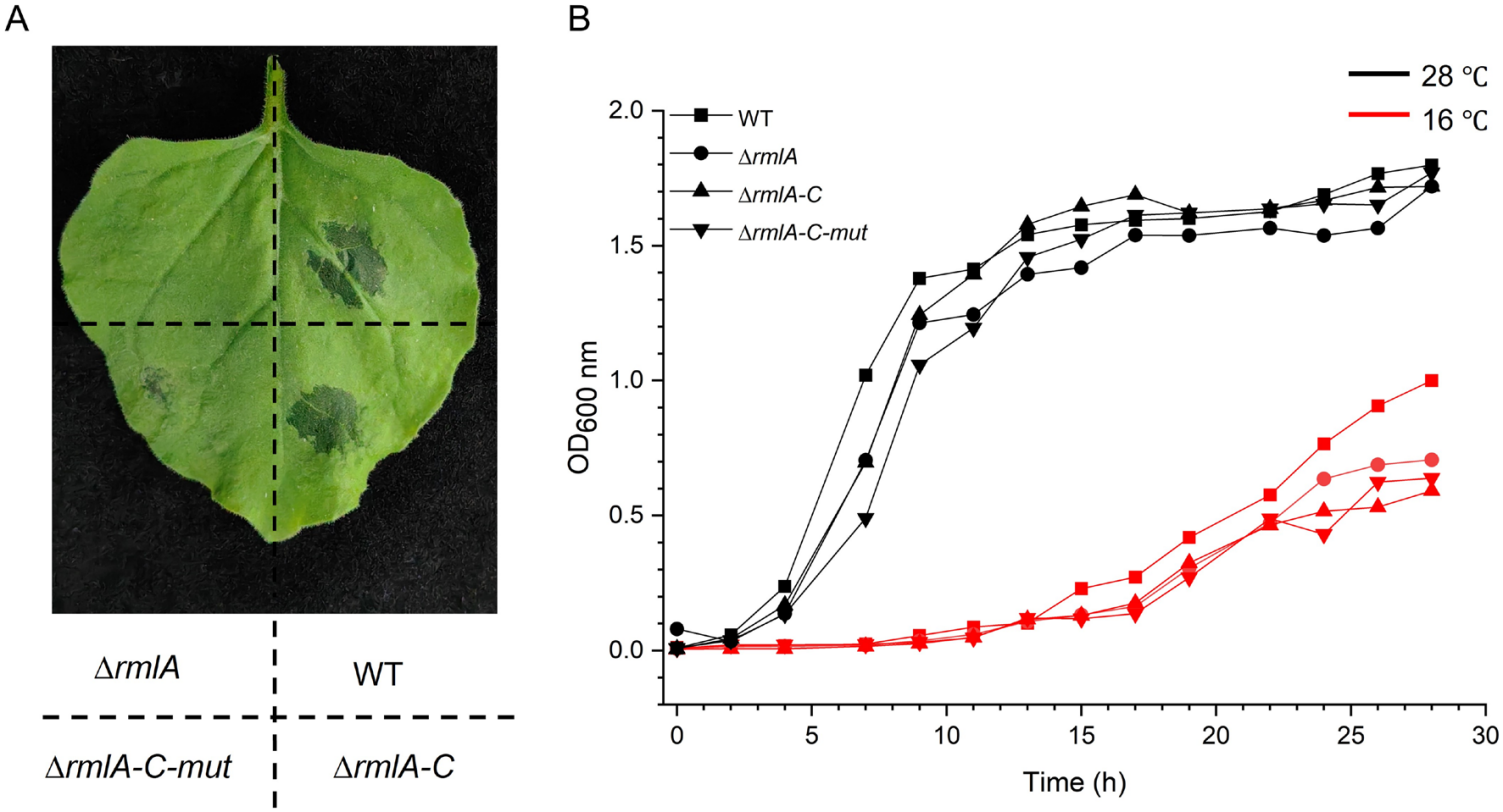
Induction of cell death by *Pectobacterium actinidiae* GX1 and determination of its growth curve. (A) To assess the ability of the *∆rmlA*, wild‐type (WT), *∆rmlA‐C* and *∆rmlA‐C‐mut* strains to induce *Nicotiana benthamiana* cell death, the strains were grown to the logarithmic growth phase, sterile water was used to resuspend the bacteria to OD_600_ = 0.3, and 7‐week‐old *N. benthamiana* leaves were infiltrated with the bacterial suspensions. After 16 h, cell death was observed and recorded. (B) For growth curve analysis of the *∆rmlA*, WT, *∆rmlA‐C* and *∆rmlA‐C‐mut* strains, the strains were grown to the logarithmic growth phase and then inoculated into 200 mL of Luria Bertani liquid medium at a ratio of 1:200. The culture was incubated in a shaker at 28°C or 16°C, and the culture was taken every 2 h to measure the OD_600_ value.

**FIGURE 7:**
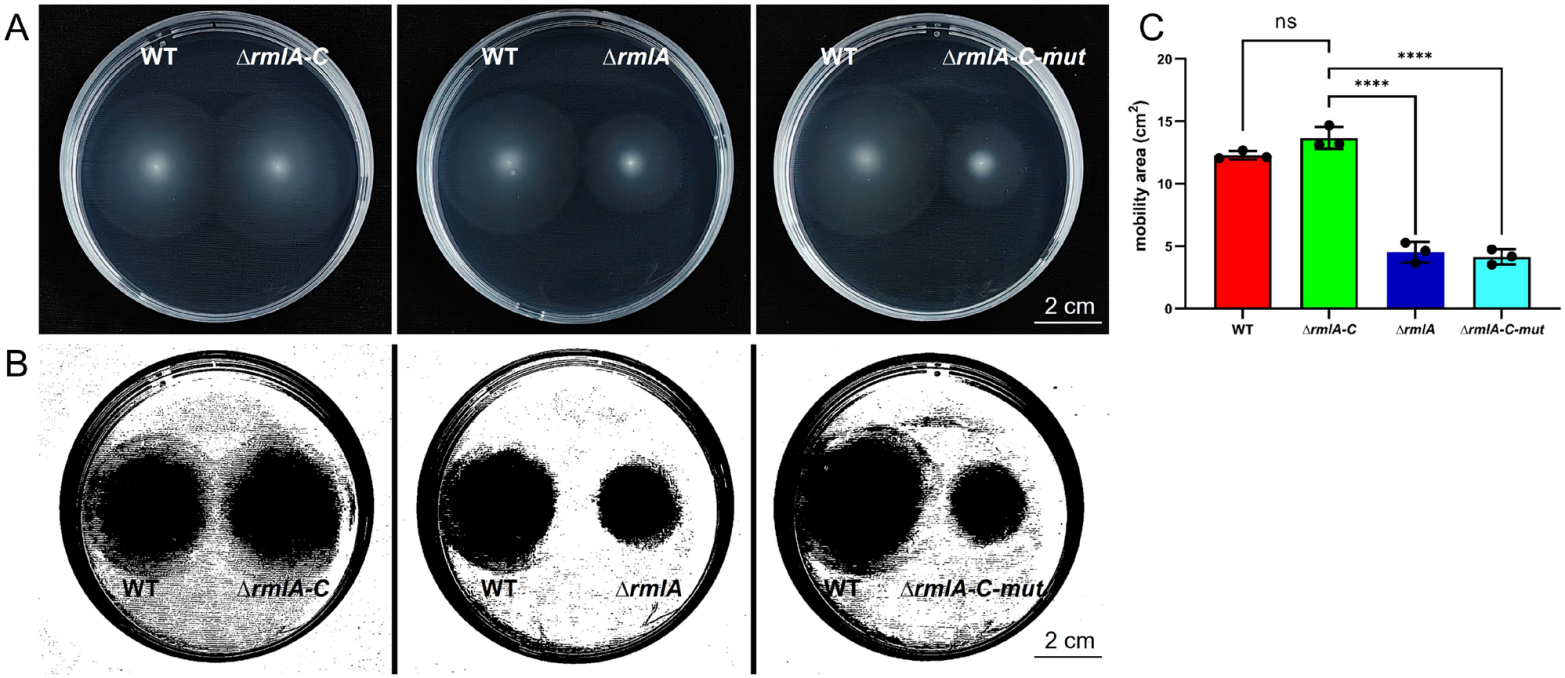
Changes in the motility of different *Pectobacterium actinidiae* GX1 mutants. (A) Assessment of the mobility of the *∆rmlA*, wild‐type (WT), *∆rmlA‐C* and *∆rmlA‐C‐mut* strains on agar plates. (B) Mobility area analysed by ImageJ in (A). (C) Bacterial mobility area corresponding to the assay data in (B). The error bars represent the standard deviation; *n* = 3 plates. Significant differences from ANOVA are shown: **** = *p *< 0.0001. The control is the motility area of WT GX1. The experiment was repeated three times, and the results all showed the same trend.

**FIGURE 8:**
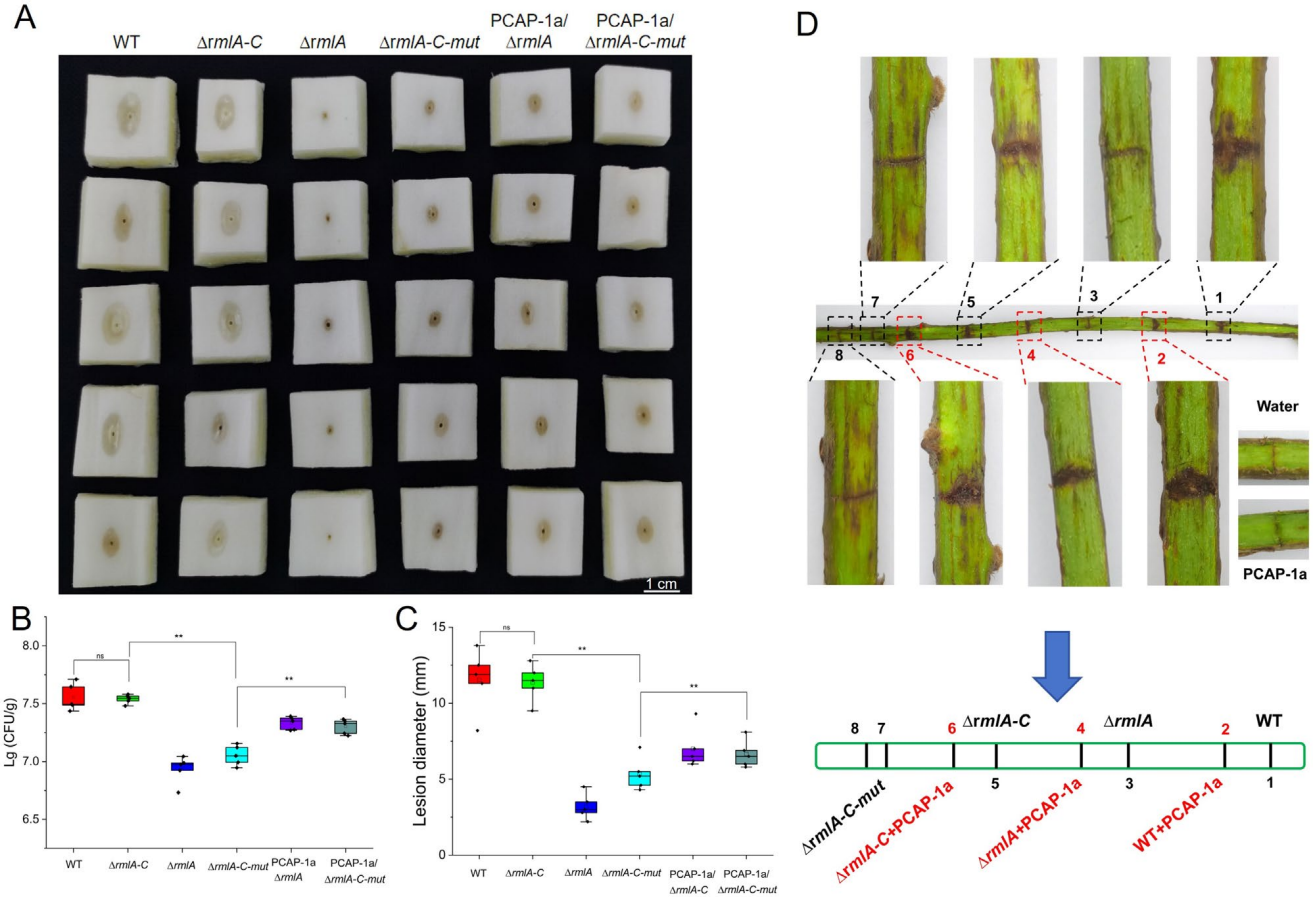
Effects of RmlA substrate‐binding capacity on the pathogenicity of *Pectobacterium actinidiae* GX1. (A) Assessment of the pathogenicity of the *∆rmlA‐C‐mut* strain in Chinese cabbage. (B) Lesion diameters corresponding to the assay data in (A). (C) Bacterial colonisation corresponding to the assay data in (A). The error bars represent the standard deviation; *n* = 5 cabbage stems. Significant differences from ANOVA are shown: ** = *p *< 0.01; ns indicates no significant difference. The control consisted of Chinese cabbages incubated with wild‐type GX1 (WT). The experiment was repeated three times, and the results all showed the same trend. (D) Pathogenicity testing of the WT and mutant strains on kiwifruit branches.

To investigate the impact of the RmlA substrate‐binding ability on host strain virulence, we complemented the *rmlA* knockout strain *∆rmlA* with a mutated version of *rmlA*. Genomic DNA from the GX1 strain was used as the template to amplify the target gene with homologous arms. To introduce the mutation, overlap extension PCR was performed to mutate the original target gene. The mutated gene was then cloned and inserted into the pBBR1‐MCS5 vector via homologous recombination. The recombinant plasmid was transformed into *Escherichia coli* S17‐λpir, which served as the donor strain. Positive transformants were selected and used for biparental mating with the ∆*rmlA* strain to introduce the mutated *rmlA* gene. The resulting ∆*rmlA‐C‐mut* strain was verified by molecular techniques and stored for future use. Unlike *∆rmlA‐C*, *∆rmlA‐C‐mut* exhibited a substantially reduced ability to induce cell death, with no evident *N. benthamiana* cell death observed following infiltration with its bacterial suspension (Figure 6A). Furthermore, to elucidate the effect of RmlA on the growth of the strains, growth curves of the WT and mutant strains were generated after culture at 28°C and 16°C. Consistent with those in the WT strain, mutations or complemented strains in RmlA did not impact strain growth (Figure 6B). However, the bacterial mobility of the *∆rmlA* and *∆rmlA‐C‐mut* strains was significantly impaired compared with that of the WT and *∆rmlA‐C* strains; mutations in RmlA notably affect swarming behaviour, resulting in reduced movement across agar plates (Figure 7A–C). Pathogenicity tests on Chinese cabbage and kiwifruit branches were conducted to evaluate the virulence of the *∆rmlA*‐*C‐mut* strain (Figure 8A–D). The diminished substrate binding ability of RmlA significantly reduced the pathogenicity of GX1 (Figure 8A). Compared with the *∆rmlA*‐*C* strain, the *∆rmlA‐C‐mut* strain produced only slightly increases in lesion diameter and colonisation compared with those of the *∆rmlA* group (Figure 8B,C). Importantly, the application of PCAP‐1a partially restored the pathogenicity of the mutated strains (Figure 8D).

## 3 Discussion

In‐depth genomic analysis was used to explore the functionality and evolution of *P. actinidiae* GX1. Parameters such as genome size, gene content and GC content are vital for interpreting a strain's evolutionary adaptations (Li et al. 2023). Interestingly, analysis of the EPS synthesis gene homology within the genus *Pectobacterium* revealed a high degree of similarity in the EPS synthesis gene distribution patterns between *P. carotovorum* and *P. brasiliense* , analogous to the patterns observed in *P. actinidiae* GX1. Furthermore, in vitro experiments have indicated that EPSs derived from *P. carotovorum* and *P. brasiliense* can induce cell death in *N. benthamiana*, similar to the effects of PCAP‐1a. These results suggest evolutionary conservation among these species and shared structural/functional features of their EPSs.

EPSs are high‐molecular‐weight polysaccharide compounds synthesised intracellularly and secreted extracellularly by bacteria during growth (Wang et al. 2021). The presence of EPSs is critical for bacterial survival and function, because EPSs not only facilitate cell adhesion and protect against harsh environments, desiccation and antimicrobial agents but also enhance bacterial adaptability, sustained colonisation, stress tolerance (Arayes et al. 2023). Pathogen‐derived EPSs often act as virulence factors that promote infection during the pathogenic process (Yuan et al. 2024). Instances of such EPSs from several plant pathogens facilitating infection have been documented, including those from *R. solanacearum* , *Erwinia amylovora* , *X. campestris* and *P. syringae* (Saile et al. 1997; Islamov et al. 2021; Aslam et al. 2008). In our study, gene knockout experiments demonstrated that PCAP‐1a enhanced host infection and is a key virulence factor in GX1 pathogenicity. Strains lacking PCAP‐1a synthesis showed nearly complete loss of pathogenicity.

In contrast to proteins directly encoded by genes, the synthesis pathways of bacterial EPSs are highly complex and often involve a variable number of genes ranging from several to dozens (Sun and Zhang 2021). These include polysaccharide transport proteins (such as WecA, Wza and Wzc), glucose‐1‐phosphate thymidylyltransferases, dTDP‐glucose‐4,6‐dehydratases and glycosyltransferases (Saulnier et al. 2011). The coordinated action of these enzymes and regulatory factors governs EPS biosynthesis and structure, ultimately shaping the pathogen's virulence. Advancements in molecular biology techniques have allowed us to directly assess the contributions of specific genes to overall pathogenicity. In this study, genomic analysis revealed that the synthesis of the GX1 EPS is controlled by a cluster of 21 genes, and through knockout experiments, we confirmed the critical role of RmlA in GX1 polysaccharide synthesis and pathogenicity. The *rmlA* gene primarily catalyses the transformation of G‐1‐P and dTTP into dTDP‐glucose, a common precursor in bacterial carbohydrate metabolism pathways that serves as a precursor for deoxyglucose (Harathi et al. 2016; Zheng et al. 2022). In many bacteria, these deoxy sugars are crucial components of EPSs, lipopolysaccharides and other glycans in the cell wall (Azimi et al. 2020; Hirmondó et al. 2022; Silva et al. 2005). Knockout of *rmlA* significantly altered the production and activity of PCAP‐1a, leading to a marked reduction in the virulence of GX1. Given the conserved catalytic activity of RmlA, we mutated the substrate binding site of RmlA, which substantially reduced the binding affinity of the RmlA substrates G‐1‐P and dTTP. More importantly, the decreased substrate binding capacity of the mutated RmlA directly led to a reduction in the pathogenicity of the GX1 strain. Collectively, our data establish RmlA as a key determinant of GX1 pathogenesis.

In this study, we conducted a comprehensive analysis of the pathogenicity of the *P. actinidiae* GX1 strain, which included assessments of the infection‐promoting effects of EPSs, knockout of the polysaccharide synthesis gene *rmlA* and analysis of the substrate activity of its encoded protein. In summary, our research confirmed that the EPS PCAP‐1a of GX1 acts as a virulence factor facilitating pathogen invasion and that the synthesis and structural integrity of PCAP‐1a are dependent on the polysaccharide synthesis gene *rmlA* and the substrate‐binding activity of its encoded protein. This study not only delineates the genomic characteristics of *P. actinidiae* GX1 but also reveals the role of bacterial EPSs and their associated biosynthetic genes in the pathogenesis of the pathogen. Furthermore, this study provides an experimental foundation and theoretical rationale for the future development of inhibitors that target the active site of RmlA.

## 4 Materials and Methods

### 4.1 Microbial Strains, Plants and Growth Conditions

The strains and plasmids used in this work are listed in Table S1, and all primers used are listed in Table S2. *P. actinidiae* GX1 was isolated from the symptomatic vines of kiwifruit cv. Hong Yang in the town of Zhongfeng, city of Guilin, China (Zhang et al. 2023), and individual colonies of *P. actinidiae* GX1 were subcultured on Luria Bertani (LB) plates to obtain pure colonies. *N. benthamiana* plants were grown in a greenhouse at 21°C–25°C under a 16‐h light/8‐h dark cycle, and 7‐week‐old plants were used for the experiments (Bai et al. 2023). *Brassica rapa* subsp. *pekinensis* is commercially available.

### 4.2 Whole‐Genome Sequencing, Assembly and Analysis

The sequencing of the *P. actinidiae* GX1 strain was commissioned to BGI‐Genomics and followed the standard protocol provided by PacBio, encompassing sample quality assessment, library construction, library quality evaluation and sequencing. Assembly was conducted using Hifiasm software, with circularisation and adjustment of the start site executed via Circlator v. 1.5.5 software. Second‐generation data were further used for error correction with Pilon v. 1.22 software to obtain a genome with enhanced accuracy for subsequent analysis (Cheng et al. 2021). Gene islands in the bacterial genome were predicted using IslandPath‐DIMOB software (Bertelli and Brinkman 2018). The protein sequences encoded by the genes were aligned with proteins in the KEGG database using BLAST to find the closest matches (Kanehisa et al. 2004). Blast2GO software was used for GO annotation of the genome‐encoded proteins (Ashburner et al. 2000).

### 4.3 GX1 Pathogenicity Determination

After GX1 was cultured with shaking, the bacteria were resuspended in sterile water until the OD_600_ reached 0.4, after which an equal volume of sterile water or PCAP‐1a solution was added for subsequent use. The core of a Chinese cabbage was cut into 2 × 2 cm^2^ pieces and inoculated with 1.5 μL of the bacterial suspension using a pipette tip. After sealing and moistening, the samples were placed in a 28°C incubator for 18 h, the lesion diameters were recorded, and dilutions from the affected areas were spread on plates for analysis. WT, mutant or complemented strains with GFP fluorescence were cultured with shaking, resuspended in sterile water until the OD_600_ reached 1.0, and then mixed with an equal volume of sterile water or PCAP‐1a solution for subsequent use. The suspension was supplemented with the surfactant Silwet L‐77 (0.03%) and then uniformly sprayed onto the surface or underside of *N. benthamiana* leaves; the fluorescence signal was observed using a confocal microscope after 24 h (Rao et al. 2021). Whole leaves were briefly placed on LB plates containing rifampicin for 5–10 s and then removed, and the plates were incubated at 28°C for 24 h. The fluorescence signals of the colonies were recorded using a bioimaging system. A ring‐like incision was made on the Hong Yang kiwifruit branches using a blade, into which 20 μL of the bacterial suspension was placed. After maintaining moisture during treatment at 28°C for 7 days, the epidermal layer was scraped off with a blade and the incidence of disease was recorded.

### 4.4 Extraction and Purification of EPSs

The extraction and purification of GX1 EPSs were performed according to the methods of Yuan et al. (2024). *P. actinidiae* GX1 was precultured and inoculated in culture medium (0.8% sucrose, 0.8% glucose, 0.8% tryptone, 0.05% (NH_4_)_2_SO_4_, 0.2% K_2_HPO_4_, 0.02% KCl, 0.02% MgSO_4_). Cultures were shaken for 96 h (200 rpm, 28°C) and then cleared of cells by centrifugation (15 min, 8000 *g*, 4°C). All the supernatants were mixed with ethanol at a final concentration of 75% to precipitate the EPSs and then incubated at 4°C overnight. The precipitate was redissolved in sterile water to obtain crude EPSs. The crude polysaccharides were deproteinised by five cycles of Sevag reagent, and the residual Sevag reagent was removed by dialysis. The crude polysaccharides were then purified via a DEAE‐52 cellulose and Superdex G‐200 column (10 × 300 mm). After concentration in an ultrafiltration tube and freeze‐drying, the purified component PCAP‐1a was obtained.

### 4.5 Scanning Electron Microscopy

GX1 was cultured in liquid LB medium for 24 h with shaking, collected by centrifugation at 8000 *g* for 5 min, resuspended in 2.5% glutaraldehyde solution, and then incubated overnight at 4°C. After a second centrifugation at 8000 *g* for 5 min, the cells were resuspended three times in 0.2 M phosphate‐buffered saline (PBS, pH 7.4). A graded series of ethanol solutions (30%, 50%, 70%, 85% and 95%) and two final 100% ethanol solutions were used for resuspension, with 20 min of standing following each step. The bacterial precipitate was resuspended in isoamyl acetate, allowed to stand overnight at 4°C, and then freeze‐dried for scanning electron microscopy analysis (Zhou et al. 2022). The images revealed the surface morphology and microstructures of the sample under different accelerating voltages (3 and 5 kV), magnifications (5000× and 30,000×), working distances and signal modes. These images were suitable for detailed analysis of a sample's surface characteristics.

### 4.6 Construction of the GX1 Mutant and Complemented Strains

The genomic DNA of GX1 was isolated and purified according to the method described by Nikodinoska et al. (2022). The primers Pa_1774‐1F, Pa_1774‐1R, Pa_1774‐3F and Pa_1774‐3R were used to amplify 600 bp segments upstream (*rmlA* ^*up*^) and downstream (*rmlA* ^*down*^) of rmlA from the GX1 genomic DNA. A *Km* fragment with LOX sites was amplified from the pET30a plasmid using the primers Pa_Km‐2F and Pa_Km‐2R. The purified fragments (*rmlA* ^*up*^, *rmlA* ^*down*^ and *Km*) were used as templates to amplify the *rmlA* ^*up*^+*rmlA* ^*down*^+*Km* segment with the primers Pa_1774‐1F and Pa_1774‐3R. The pEX18Gm plasmid was digested with KpnI and XbaI, and the resulting products were ligated into the *rmlA* ^*up*^+*rmlA* ^*down*^+*Km* segment. The ligation product was transformed into *E. coli* S17‐λpir competent cells. Recombinant plasmid containing *E. coli* S17‐λpir was mixed with GX1 in equal proportions. A sterile 0.22 μm filter was placed on an LB agar plate, and 50 μL of the bacterial mixture was spotted onto the filter. After air drying in a laminar flow hood, the plate was incubated at 28°C for 24 h to facilitate biparental mating. The bacterial plaques on the filter were eluted and resuspended in an appropriate volume of sterile water, followed by suitable dilution. Then, 100 μL of the diluted suspension was spread onto LB plates containing kanamycin, rifampicin and 15% sucrose. After incubation at 28°C for 48 h, single colonies were picked for colony PCR verification. The sequencing results were subsequently used to confirm the presence of double crossovers (Yamada et al. 2008).

*Km* resistance was determined via the following steps. First, *E. coli* S17‐λpir harbouring the pEX18‐Cre plasmid was precultured in liquid LB medium supplemented with gentamicin. This strain was then mixed in equal proportions with the GX1 double‐crossover mutant for biparental mating. After colony development, the colonies were screened using the primers Pa_1774‐YZ‐F1 and Pa_1774‐YZ‐R2 for PCR verification. The strains confirmed via PCR were sequentially cultured with shaking for 12 h in liquid LB medium supplemented with rifampicin, rifampicin plus 15% sucrose and rifampicin again to completely eliminate the pEX18‐Cre plasmid. The *Km* fragment was removed from the resulting mutant strain (∆*rmlA*), which retained only rifampicin resistance. The target gene *rmlA* (with its own promoter fragment) was amplified using the primers Pa_1774‐pBBR‐F and Pa_1774‐pBBR‐R, with GX1 DNA used as the template. After double enzyme digestion and purification, the pBBR1‐MCS5 plasmid was recombined with the *rmlA* target gene, which contains homologous arms, and then transformed into *E. coli* DH5α competent cells. The recombinant plasmid was transformed into *E. coli* S17‐λpir competent cells. Positive transformants were verified by colony PCR. The recombinant plasmid was subsequently transformed into *∆rmlA* through biparental mating, resulting in the generation of the complemented strain *∆rmlA‐C*.

### 4.7 Protein Modelling and Molecular Docking of RmlA

The RmlA protein was modelled online using the Phyre2 website (http://www.sbg.bio.ic.ac.uk/phyre2/html/page.cgi?id=index). Three‐dimensional models of the G‐1‐P Na_2_ and dTTP Na_2_ ligands were obtained from the RCSB database (https://www.rcsb.org/structure); water molecules were manually eliminated from the RmlA model, polar hydrogen atoms were added using Discovery Studio software, and molecular docking simulations were performed with the respective ligands to generate visual 3D and 2D models.

### 4.8 Prokaryotic Expression

The target gene containing the homologous arms of pET32a was obtained using GX1 genomic DNA as a template. To obtain the mutated target gene, the original target gene was mutated using the overlap extension PCR. The intact *rmlA* or mutated *rmlA* target gene was inserted into the prokaryotic expression vector pET32a through homologous recombination. The constructed vector was then transformed into *E. coli* ArcticExpress (DE3) host cells for low‐temperature‐induced expression. After cell lysis, a small amount of FITC (Beijing Solarbio Science & Technology Co. Ltd.) powder was added, and purification was performed according to the method described by Yamada et al. (2008). The exchange buffer used was 50 mM Tris‐HCl buffer (containing 1 mM MgCl_2_ and 0.05% Tween 20, pH 8.0).

### 4.9 MST Assay

Solutions of G‐1‐P Na_2_ and dTTP Na_2_ were prepared separately, each to a final concentration of 400 μM, in Tris‐HCl buffer (50 mM Tris‐HCl, 1 mM MgCl_2_, 0.05% Tween 20, pH 8.0). After centrifugation at 12,000 *g* for 20 min, the supernatant was collected for further analysis. An appropriate amount of the RmlA fusion protein was diluted to the desired concentration after centrifugation at 12,000 *g* for 20 min. The experimental instrument used was a Monolith NT.115. RmlA or ligands (G‐1‐P Na_2_ and dTTP Na_2_) were loaded into capillaries, and the blue channel was selected. Pretest and binding‐check assays were performed to confirm binding. The ligand solutions were subsequently diluted to generate solutions with 16 different concentrations and mixed with RmlA for binding affinity detection. MO.Affinity analysis software was used to analyse the test results and calculate the binding affinity.

### 4.10 Strains and Their Nucleotide Sequence Accession Numbers

The complete genomic information of the members of the genus *Pectobacterium* was obtained from the NCBI and included the following strains: *P. carotovorum* WPP14 (CP051652), *P. atrosepticum* 21A (CP009125), *P. brasiliense* 1692 (CP047495), *P. parmentieri* IFB5427 (CP027260.1), *P. wasabiae* GFBP 3304 (CP015750), *P. odoriferum* JK2.1 (CP034938), *P. peruviense* UGC32, *P. polaris* NIBIO1392 (CP017482) and *P. betavasculorum* NCPPB2795.

### 4.11 Swarming Motility Assays

The swarming phenotypes were assessed as previously reported for *Pseudomonas aeruginosa* (Yan, Monaco, et al. 2019). Briefly, freshly prepared plates of 0.3 × Bacto agar base (BD‐Difco), supplemented with 1% tryptone (Oxoid) and 0.5% NaCl, were inoculated with 2 μL of approximately 5 × 10^7^ cells. Once the drop was completely dry, the plates were incubated at 28°C in a humidity chamber for 24 h. Experiments were performed at least three times.

### 4.12 Graphical Processing and Statistical Analysis

All experiments were performed at least three independent replicates. We used Origin 8.0 to generate the graphs for this study, and all the data are expressed as the mean ± *SD*. All the data were evaluated by one‐way ANOVA. We used SPSS v. 25 to perform the statistical analysis, and statistical significance is indicated by *p* < 0.05 or *p* < 0.01.

## Author Contributions

**Zhixiang Yuan:** writing – original draft, investigation, methodology. **Yijie Yu:** data curation, formal analysis. **Tingmi Yang:** conceptualization. **Yuwei Xue:** software, writing – review and editing. **Jiangfei Hu:** formal analysis. **Njoroge Hellen Wambui:** methodology. **Zhuang Liu:** formal analysis. **Mingzhao Wang:** methodology. **Hongxia Liu:** project administration, supervision, resources.

## Conflicts of Interest

The authors declare no conflicts of interest.

## Supporting information


**Figure S1.** Assay on *Nicotiana benthamiana* leaf cell death induced by GX‐Pa1 mutant strains. After knocking out the mutant strain of exopolysaccharide synthesis genes, the bacterial solution was adjusted with pure water to OD_600_ = 0.3 and was infiltrated into the 7‐week‐old *N. benthamiana* leaves. The plants were subsequently cultured under greenhouse conditions for 16 h, after which cell death was observed and documented photographically.

**Figure S2.** Effect of *rmlA* knockout on the adhesion of GX‐Pa1. The fluorescent labelled ∆*rmlA*, ∆*rmlA‐C* and wild‐type (WT) strains were cultured to the logarithmic growth phase, and the pure water was used to resuspend the bacteria to OD_600_ = 1.0 and the same volume of pure water or PCAP‐1a solution was added into the bacteria suspension. The 7‐week‐old *Nicotiana benthamiana* leaves were infiltrated by spraying the above bacteria suspension. (a) The plants were subsequently cultured under normal lighting conditions for 24 h, after which the colonisation of the strains on the surface of the leaves was observed under the fluorescent microscope, the scale bar is 20 μm. (b) The complete leaves were attached to the LB solid medium containing Rif for 8–10 s. After the plate was incubated at 28°C for 24 h, the fluorescence intensity was observed in the bioimager.

**Figure S3.** Bacterial colonisation levels corresponding to Figure S2. 0.2 g of leaf tissue was weighed and ground uniformly in a mortar. After gradient dilution, the sample was plated onto LB solid medium containing Rif. Colony counts were recorded after 24 h. The experiment was repeated three times. ANOVA was performed, with ‘ns’ indicating no statistical difference, ‘*’ representing significance (*p* < 0.05), and ‘**’ indicating high significance (*p* < 0.01).

**Figure S4.** Characterisation of RmlA and its mutants. (a) Amino acid sequences of RmlA and its mutants; (b) SDS–PAGE of purified RmlA and its mutant proteins.

**Table S1.** The strains and plasmids used in this work.

**Table S2.** The primer sequences used in this work.

## Data Availability

All data are contained within the manuscript, figures and Supporting Information.
