# Supplementary material for: The Enzyme Glucose‐1‐Phosphate Thymidylyltransferase RmlA Plays a Crucial Role in the Pathogenesis of Pectobacterium actinidiae GX1
Source: Mol Plant Pathol. 2025 Jul 4;26(7):e70118. doi: 10.1111/mpp.70118 (PMC12227328; doi:10.1111/mpp.70118)
Supplement: Supplementary file 1 — Figure S1. Assay on Nicotiana benthamiana leaf cell death induced by GX‐Pa1 mutant strains. After knocking out the mutant strain of exopolysaccharide synthesis genes, the bacterial solution was adjusted with pure water to OD600 = 0.3 and was infiltrated into the 7‐week‐old N. benthamiana leaves. The plants were subsequently cultured under greenhouse conditions for 16 h, after which cell death was observed and documented photographically. [file MPP-26-e70118-s004.docx]

**Supplementary Figures**


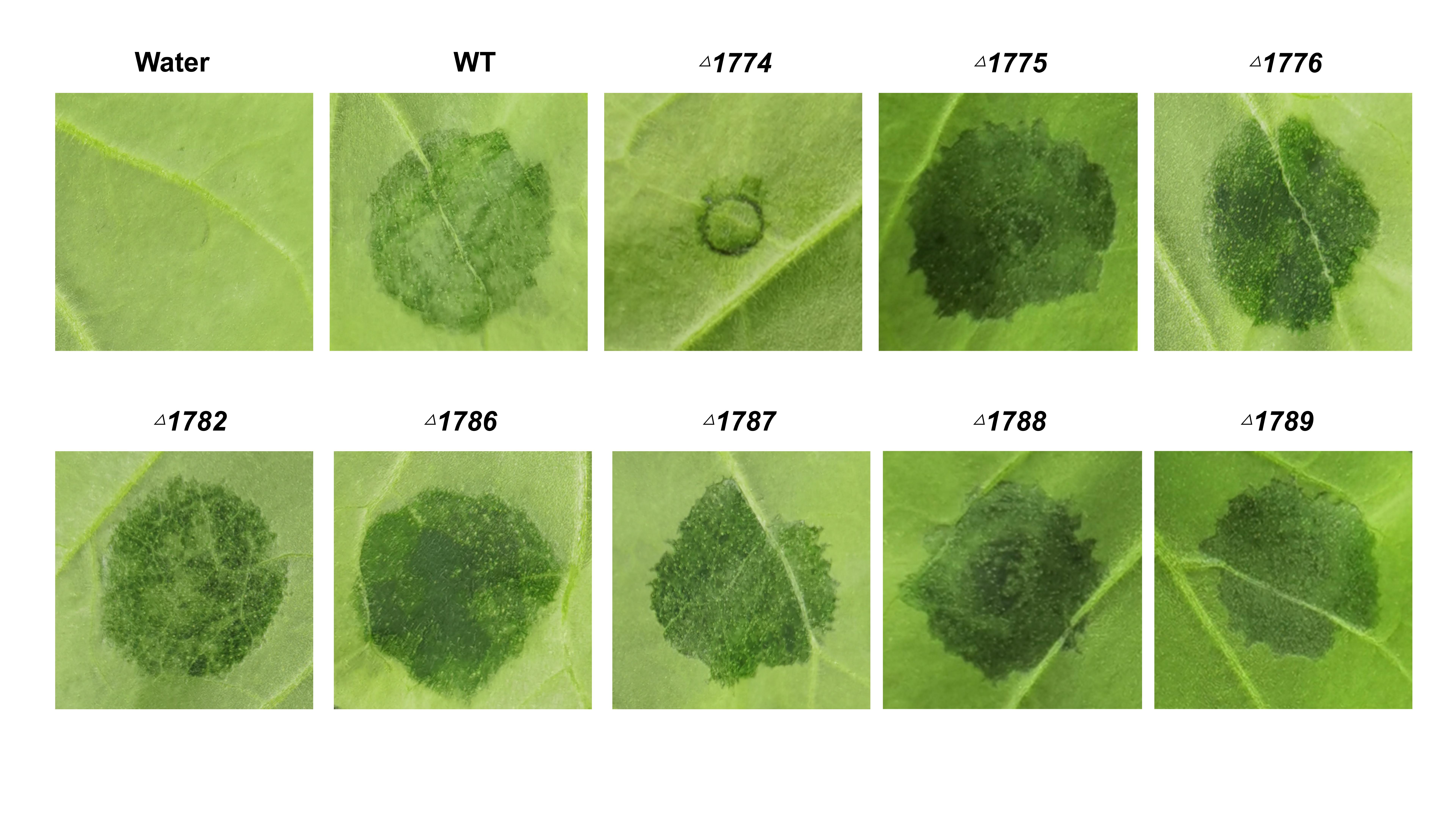


**Figure. S1 Assay on *N. benthamiana* leaf cell death induced by GX-Pa1 mutant strains**

After knocking out the mutant strain of exopolysaccharide synthesis genes, the bacterial solution was adjusted with pure water to OD_600_=0.3, and was infiltrated into the 7-week-old *N. benthamiana* leaves. The plants were subsequently cultured under greenhouse conditions for 16 hours, after which cell death was observed and documented photographically.
