# Supplementary material for: The Enzyme Glucose‐1‐Phosphate Thymidylyltransferase RmlA Plays a Crucial Role in the Pathogenesis of Pectobacterium actinidiae GX1
Source: Mol Plant Pathol. 2025 Jul 4;26(7):e70118. doi: 10.1111/mpp.70118 (PMC12227328; doi:10.1111/mpp.70118)
Supplement: Supplementary file 2 — Figure S2. Effect of rmlA knockout on the adhesion of GX‐Pa1. The fluorescent labelled ∆rmlA, ∆rmlA‐C and wild‐type (WT) strains were cultured to the logarithmic growth phase, and the pure water was used to resuspend the bacteria to OD600 = 1.0 and the same volume of pure water or PCAP‐1a solution was added into the bacteria suspension. The 7‐week‐old Nicotiana benthamiana leaves were infiltrated by spraying the above bacteria suspension. (a) The plants were subsequently cultured under normal lighting conditions for 24 h, after which the colonisation of the strains on the surface of the leaves was observed under the fluorescent microscope, the scale bar is 20 μm. (b) The complete leaves were attached to the LB solid medium containing Rif for 8–10 s. After the plate was incubated at 28°C for 24 h, the fluorescence intensity was observed in the bioimager. [file MPP-26-e70118-s002.docx]

**Supplementary Figures**


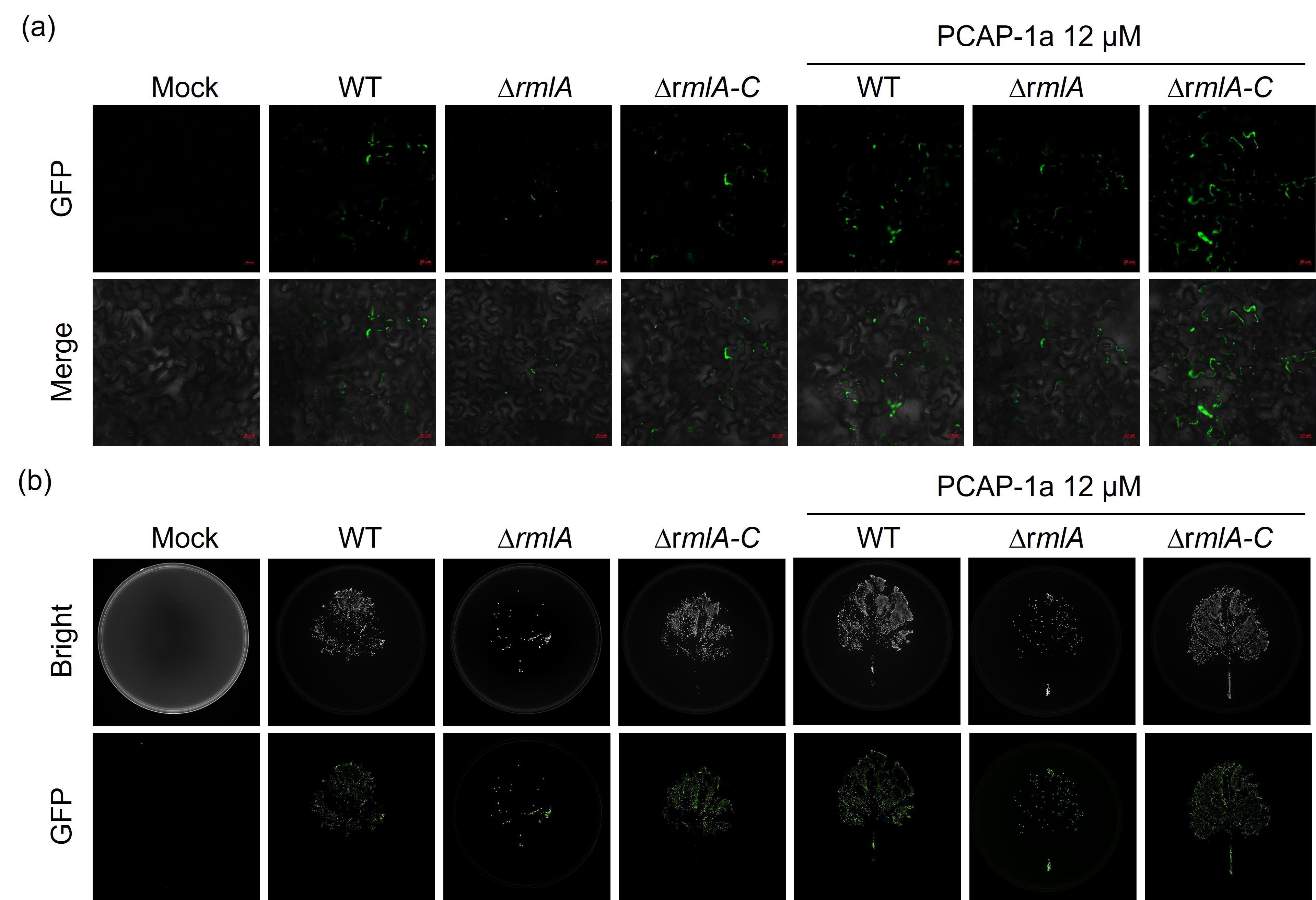


**Figure S2 Effect of *rmlA* knockout on the adhesion of GX-Pa1**

The fluorescent labeled *∆ rmlA*, *∆ rmlA-C* and WT strains were cultured to the logarithmic growth phase, and the pure water was used to resuspend the bacteria to OD_600_=1.0, and the same volume of pure water or PCAP-1a solution was added into the bacteria suspension. The 7-week-old *N. benthamiana* leaves were inoculated by spraying the above bacteria suspension. (a)The plants were subsequently cultured under normal lighting conditions for 24 hours, after which the colonization of the strains on the surface of the leaves was observed under the fluorescent microscope, the scale bar is 20 μm. (b)The complete leaves were attached to the LB solid medium containing Rif for 8-10 seconds. After the plate was incubated at 28 °C for 24 hours, the fluorescence intensity was observed in the bioimager.
