# Supplementary material for: The Enzyme Glucose‐1‐Phosphate Thymidylyltransferase RmlA Plays a Crucial Role in the Pathogenesis of Pectobacterium actinidiae GX1
Source: Mol Plant Pathol. 2025 Jul 4;26(7):e70118. doi: 10.1111/mpp.70118 (PMC12227328; doi:10.1111/mpp.70118)
Supplement: Supplementary file 3 — Figure S3. Bacterial colonisation levels corresponding to Figure S2. 0.2 g of leaf tissue was weighed and ground uniformly in a mortar. After gradient dilution, the sample was plated onto LB solid medium containing Rif. Colony counts were recorded after 24 h. The experiment was repeated three times. ANOVA was performed, with ‘ns’ indicating no statistical difference, ‘*’ representing significance (p < 0.05), and ‘**’ indicating high significance (p < 0.01). [file MPP-26-e70118-s001.docx]

**Supplementary Figures**





**Figure S3 Bacterial colonization levels corresponding to Figure S2.**

0.2 g of leaf tissue was weighed and ground uniformly in a mortar. After gradient dilution, the sample was plated onto LB solid medium containing Rif. Colony counts were recorded after 24 hours. The experiment was repeated three times. ANOVA was performed, with "ns" indicating no statistical difference, "*" representing significance (P<0.05), and "**" indicating high significance (P<0.01).
