# Supplementary material for: The Enzyme Glucose‐1‐Phosphate Thymidylyltransferase RmlA Plays a Crucial Role in the Pathogenesis of Pectobacterium actinidiae GX1
Source: Mol Plant Pathol. 2025 Jul 4;26(7):e70118. doi: 10.1111/mpp.70118 (PMC12227328; doi:10.1111/mpp.70118)
Supplement: Supplementary file 4 — Figure S4. Characterisation of RmlA and its mutants. (a) Amino acid sequences of RmlA and its mutants; (b) SDS–PAGE of purified RmlA and its mutant proteins. [file MPP-26-e70118-s003.docx]

**Supplementary Figures**


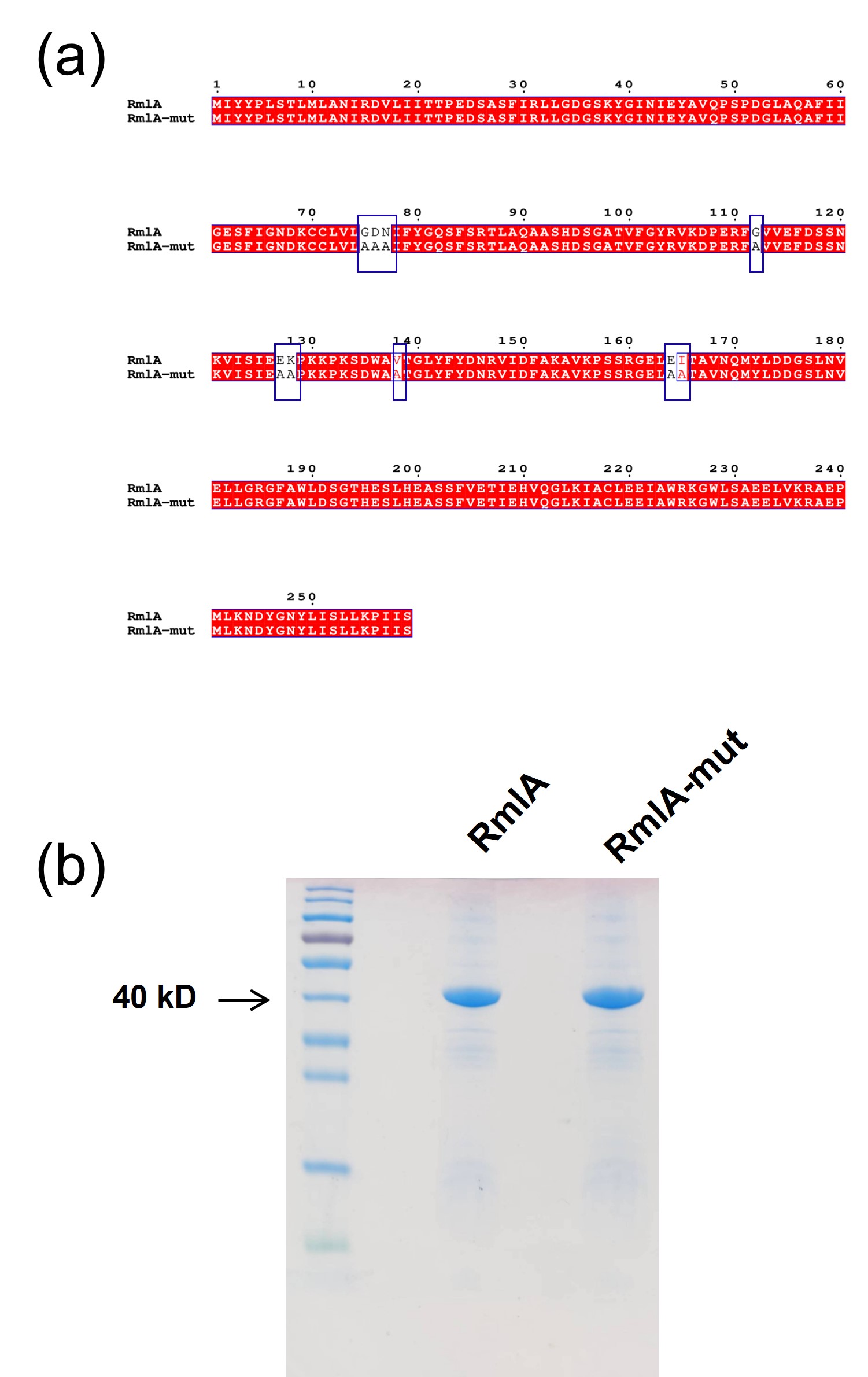


**Figure S4 Characterization of RmlA and its mutants**

(a) Amino acid sequences of RmlA and its mutants;

(b) SDS‒PAGE of purified RmlA and its mutant proteins;
