# Supplementary material for: The Enzyme Glucose‐1‐Phosphate Thymidylyltransferase RmlA Plays a Crucial Role in the Pathogenesis of Pectobacterium actinidiae GX1
Source: Mol Plant Pathol. 2025 Jul 4;26(7):e70118. doi: 10.1111/mpp.70118 (PMC12227328; doi:10.1111/mpp.70118)
Supplement: Supplementary file 5 — Table S1. The strains and plasmids used in this work. [file MPP-26-e70118-s005.docx]

**Table S1**

The strains and plasmids used in this work.

| Strains/plasmids | Relevant characteristics | Source |
| --- | --- | --- |
| *P. actinidiae* GX1 | | |
| WT | *P. actinidiae* GX1 Wild type Rif | Lab collection |
| *∆Pa_1774* | *Pa_1774* knockout mutant Rif | This study |
| *∆rmlA* | *rmlA*(*Pa_1774*) knockout mutant Rif | This study |
| *∆Pa_1775* | *Pa_1775* knockout mutant Rif | This study |
| *∆Pa_1776* | *Pa_1776* knockout mutant Rif | This study |
| *∆Pa_1782* | *Pa_1782* knockout mutant Rif | This study |
| *∆Pa_1786* | *Pa_1786* knockout mutant Rif | This study |
| *∆Pa_1787* | *Pa_1787* knockout mutant Rif | This study |
| *∆Pa_1788* | *Pa_1788* knockout mutant Rif | This study |
| *∆Pa_1789* | *Pa_1789* knockout mutant Rif | This study |
| *∆rmlA-C* | *∆rmlA* complemented with intact *Pa_rmlA* gene Rif Gm | This study |
| *∆rmlA-C-mut* | *∆rmlA* complemented with mutant *Pa_rmlA* gene Rif Gm | This study |
| WT-GFP | WT with *gfp* gene Rif Km | This study |
| *∆rmlA-*GFP | *∆rmlA* with gfp gene Rif Gm Km | This study |
| *∆rmlA-C-*GFP | *∆rmlA-C* with gfp gene Rif Gm Km | This study |
| *Escherichia coli* | | |
| DH5α | Δ(lacZYA-argF)U169, deoR, recA1, endA1 | Lab collection |
| S17-λpir | LAMpir, recA1, endA1, thiE1, hsdR17 | Lab collection |
| DH5α-GFP | DH5α with gfp gene Km | Lab collection |
| Plasmids | | |
| pET30a | expression vector, Km | Lab collection |
| pEX18Gm | Allelic exchange suicide vector, Gm | Lab collection |
| pBBR1 MCS-5 | Broad-host-range cosmid vector, Gm | Lab collection |
| pET32a | expression vector, Amp | Lab collection |
| pEX18-Cre | Gm | Lab collection |
