# Supplementary material for: The Enzyme Glucose‐1‐Phosphate Thymidylyltransferase RmlA Plays a Crucial Role in the Pathogenesis of Pectobacterium actinidiae GX1
Source: Mol Plant Pathol. 2025 Jul 4;26(7):e70118. doi: 10.1111/mpp.70118 (PMC12227328; doi:10.1111/mpp.70118)
Supplement: Supplementary file 6 — Table S2. The primer sequences used in this work. [file MPP-26-e70118-s006.docx]

**Table S2**

The primer sequences used in this work.

| Primer | Sequence |
| --- | --- |
| *Pa_1774-*1F | TACGAATTCGAGCTCGGTACCCGGGAACATCTCTTCTGGTTGC |
| *Pa_1774-*3R | TGCCTGCAGGTCGACTCTAGATTTCAGCGATAAGGATTAATGCATCAGC |
| *Pa_Km-*2F | TACCGTTCGTATAGCATACATTATACGAAGTTATGAAGCTCCCTCGTGC |
| *Pa_Km*2R | TACCGTTCGTATAATGTATGCTATACGAAGTTATCAGGTGGCACTTTTCG |
| *Pa_1774-*3F | GCTATACGAACGGTATGGCTTGTCATAAACAGGCAACA |
| *Pa_1774-*1R | ATTATACGAACGGTAGAGCCAAAATGAAAATATTGATTACAGG |
| *pEX18-TY-*F | TGTGGAATTGTGAGCGGATAACAATTTCAC |
| *Pa_1774-pBBR-*F | TTCCTGCAGCCCGGGGGATCCGTAGTTATATAGCGCCTGCTGAG |
| *Pa_1774-pBBR-R* | CGCGGTGGCGGCCGCTCTAGAAGAAATAATAGGTTTAAGCAAAGATATTAG |
| *Pa_1774-pET32a-*F | GCCATGGCTGATATCGGATCCATGATTTATTACCCGCTGTCGA |
| *Pa_1774-pET32a-*R | GTGGTGGTGGTGGTGCTCGAGAGAAATAATAGGTTTAAGCAAAGATATTAG |
| *Pa_1774-mut757677-*F | GTTGTTTAGTTCTCGCCGCTGCTATTTTCTATGGACA |
| *Pa_1774-mut757677-*R | TGTCCATAGAAAATAGCAGCGGCGAGAACTAAACAAC |
| *Pa_1774-mut112-*F | AGAACGTTTTGCTGTTGTAGAGTT |
| *Pa_1774-mut112-R* | AACTCTACAACAGCAAAACGTTCT |
| *Pa_1774-mut127128-*F | GATTTCTATCGAAGCAGCACCCAAAAAACCA |
| *Pa_1774-mut127128-*R | TGGTTTTTTGGGTGCTGCTTCGATAGAAATC |
| *Pa_1774-mut138-*F | ATTGGGCAGCTACTGGGCTTTA |
| *Pa_1774-mut138-*R | TAAAGCCCAGTAGCTGCCCAAT |
| *Pa_1774-mut164165-*F | TGAGCTTGCAGCCACAGCTGTGAATCAAAT |
| *Pa_1774-mut164165-*R | ATTTGATTCACAGCTGTGGCTGCAAGCTCA |
| *pBBR1-MCS5-TY-*F | CAGGAAACAGCTATGACC |
| *pBBR1-MCS5-TY-*R | TGTAAAACGACGGCCAGT |
| *Pa_1774-YZ-*F1 | TTGGCTGTGACGGATGCTG |
| *Pa_1774-YZ-*R1 | ATTTAATCGCGGCCTAGAGCA |
| *Pa_1774-YZ-*F2 | TTACTCACCACTGCGATCCCC |
| *Pa_1774-YZ-*R2 | CACTAAAATGAAAAATGCCCCATGC |
